# Percutaneous ruminostomy guided by rumenoscopy: study in an experimental model in bovine fetus

**DOI:** 10.1186/s12917-022-03143-5

**Published:** 2022-01-17

**Authors:** Gabriela Melo Alves dos Santos, Luisa Pucci Bueno Borges, Hanna Lyce Magno de Morais, Barbara da Conceição Guilherme, Rodrigo dos Santos Albuquerque, Kayan Cunha Rossy, Heytor Jales Gurgel, Camila do Espirito Santo Fernandes, João Pedro Monteiro Barroso, Priscila do Santos Ribas, Francisco Décio de Oliveira Monteiro, Chayanne Silva Ferreira, Pedro Paulo Maia Teixeira

**Affiliations:** 1 Veterinary Hospital, Veterinary Institute, Federal University of Pará (HV/IMV/UFPA) Castanhal Campus II, Br 316, Km 62, Castanhal, PA 68743-970 Brazil; 2 University of Rio Verde (UNIRV), Rio Verde, Goiás, Brazil

**Keywords:** Ruminants, Rumen surgery, Rumen cannulation, Endoscopy

## Abstract

**Background:**

Endosurgery is a surgical subspecialty that has been widely used in production animals, because it enables good visualization of abdominal organs and the diagnosis and treatment of several conditions in a minimally invasive manner, while preserving the animal’s well-being and causing a lower impact on animal production. Rumenostomy is one of the most common surgical procedures in ruminants. This procedure is used to allow access to the rumen for various purposes, especially nutritional and therapeutic studies, and it can be performed either in a conventional way or in a minimally invasive video-assisted manner. Another possibility of access to ruminants is through the rumenoscopy technique. The objective of this study is to describe a minimally invasive technique for rumenostomy using an endoscope, working on a bovine fetal corpse as an experimental model.

**Results:**

The execution of the endoscopy-guided rumenostomy technique was simple and did not present major difficulties. The endoscope, its lighting and air pump, and the decubitus used provided a good anatomical visualization of the rumen, and it was possible to evaluate several regions of the organ. The mean duration of the procedure was 11.15 min.

**Conclusions:**

The endoscopic rumenostomy technique using anatomical pieces of calves was shown to be feasible. It was performed in a simple and efficient way, particularly regarding the premise of preserving the animal’s well-being, due to its minimally invasive nature.

**Supplementary Information:**

The online version contains supplementary material available at 10.1186/s12917-022-03143-5.

## Background

Endosurgery is a surgical subspecialty that has been widely used in production animals. It has shown increasingly promising results [26] and in many cases has become the surgical technique of choice, since it allows good visualization of abdominal organs [4] and the diagnosis and treatment of several conditions in a minimally invasive manner, while preserving the animal’s well-being and causing a lower impact on animal production [34].

The advantages of endosurgery include less tissue injury, lower risk of infections and post-operative pain due to a smaller surgical incision, less exposure and manipulation of the viscera, shorter surgical time, and faster recovery for the animal. These factors should be taken into consideration when deciding the surgical technique to be used [9, 36].

Numerous surgical procedures can be performed through the endosurgical approach, including laparoscopy, which allows access, visualization, and manipulation of the abdominal cavity and its organs. Laparoscopy can be performed for various purposes, such as abomasopexy [1], kidney biopsy [7], liver biopsy [10], cystotomy [15] ovariectomy [2, 3, 34].

Rumenostomy is one of the most common surgical procedures in ruminants. It is used to open an access to the rumen, either temporarily or permanently, and has several purposes, particularly animal nutrition studies to assess digestibility and rumen metabolism and to evaluate animals that will serve as donors of ruminal inoculum [29, 32, 37], in addition to therapeutic purposes, such as cases of relapsing tympanism due to vagal indigestion [30], calves with tympanism due to esophageal groove dysfunction [22], and enteral nutrition [5].

In its traditional forms, rumenostomy has variations regarding the material used. It can use either a rigid cannula [12, 24] or a flexible one [34]. Regarding its execution, it is possible to perform it in either one or two surgical stages [27, 35]. In 2018, Santos et al. described a minimally invasive technique of video-assisted rumenostomy in sheep. In ruminants, another possibility is rumenoscopy, which through the use of an endoscope makes it possible to visualize the structures of the rumen [14, 25].

The use of animals in the study of techniques and treatments, in both veterinary and human medicine, is still questioned and much discussed. The “3R principle” (reduction, refinement, replacement), established by William Russell and Rex Burch in 1959, is most widely used by researchers, because it emphasizes the importance of developing studies that allow a reduction in the number of animals used without impacting the reliability of the results; the replacement of the use of animals by other research models, such as the use of “organs-on-a-chip,” three-dimensional and computerized tissue models, and the model used in the present study; and the refinement of techniques that have less impact on animal health [6, 18, 20].

In this same sense, the choice of minimally invasive procedures becomes increasingly advantageous and necessary, since it allows results and advances to be obtained while considering the ethics in animal experimentation and the animals’ well-being [28, 34]. Other studies also seek alternative techniques for rumen cannulation with esophageal or nasoesophageal probes, particularly in cases where it is necessary to repeat the procedure several times in the same animal [32].

Therefore, the aim of this study is to describe and standardize a minimally invasive rumenostomy technique by ororuminal endoscopy and percutaneous cannulation, working on an experimental model in bovine fetus corpses, which had been previously slaughtered by accident and would be discarded in local slaughterhouses.

## Results

The endoscopy-guided rumenostomy technique shown to be feasible and presented no difficulties or complications.

**Fig. 1:**
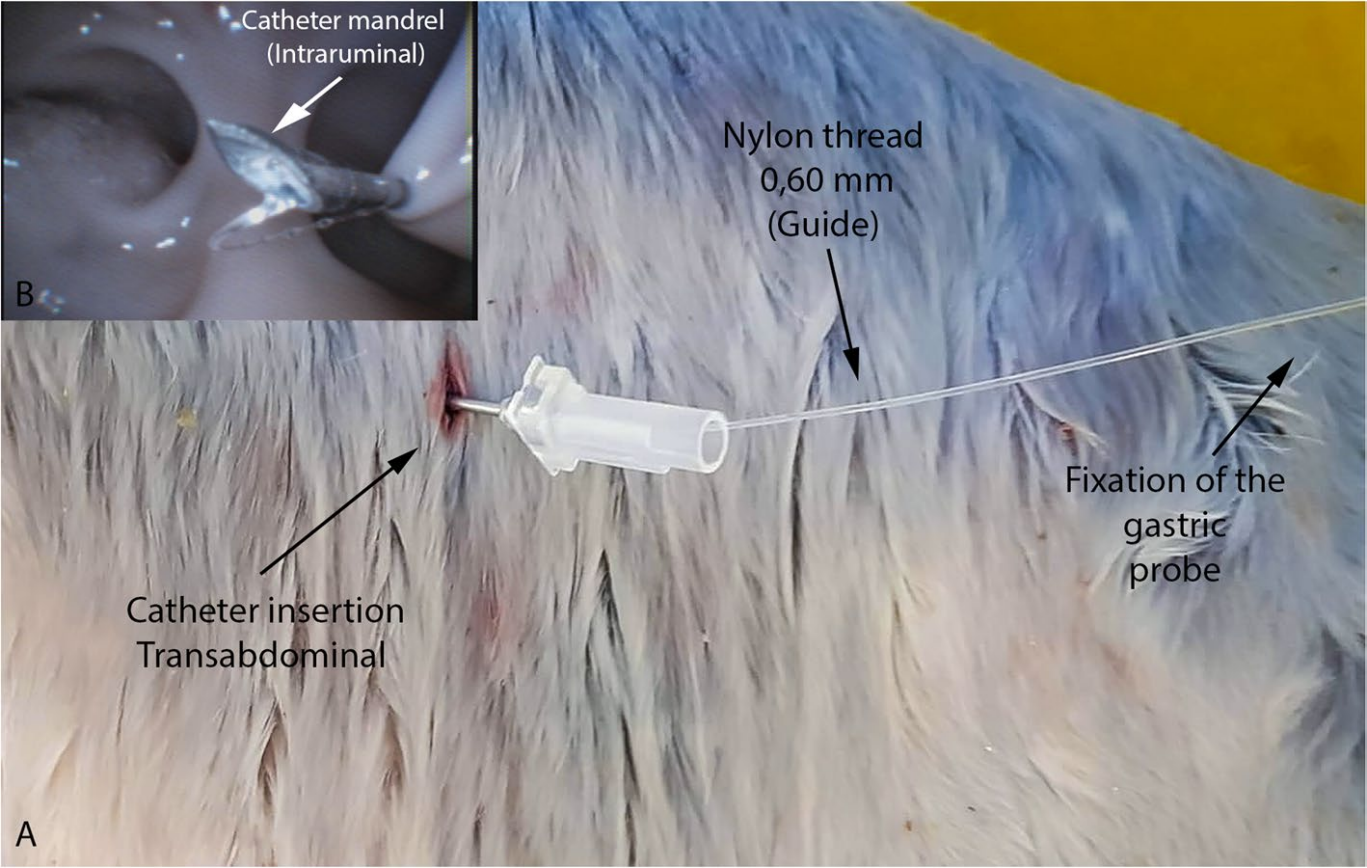
Catheter insertion transabdominal. **A** External view of the catheter and guidewire; **B** Internal view of the catheter needle and the introduction of the tube guidewire. Catheter needle

The endoscope’s air pump was sufficient to inflate the organ and allow its internal visualization. The endoscope provided a good anatomical visualization of the rumen, from its entrance from the esophagus to its internal structures, and it was possible to evaluate the dorsal and ventral sacs of the rumen, the caudodorsal and caudoventral blind sacs, the dorsal and ventral coronary pillars, and the caudal pillar (Fig. 1G).

The decubitus used, the transillumination from the endoscope, and the palpation on the flank showed to be adequate for locating and positioning the probe in the left paralumbar fossa. The catheter used for the passage of the guide probe and the endoscopic probe showed to be efficient for the objectives of this study.

The mean duration of the procedure, from the passage of the endoscope, inflation, and passage of the guide and probe to the incision and fixation of the probe, was 11.15 ± 0.7 min.

## Discussion

The technique established in this study proved to be efficient in terms of the simplicity of its approach. A technique similar to the presently described one is percutaneous endoscopic gastrostomy, performed in humans and described by Gauderer et al. in 1980, which has indications in cases of patients unable to eat normally for reasons such as neuropathies, congenital diseases, neoplasms, traumas, etc. The main advantages of both techniques are the possibility of gastrostomy without the need for laparotomy, avoiding further injuries to patients; less postoperative pain; and shorter surgical and anesthetic time.

Some minimally invasive techniques performed in the gastrointestinal tract of ruminants have been described, including laparoscopic abomasopexy, used to treat abomasum displacement, and laparoscopic abomasal cannulation [1, 19, 38].

Rumenoscopy is the visualization of the ruminal structures with an endoscope. It allows the cannulation of the rumen, as described in this study. It is believed that percutaneous rumenostomy by rumenoscopy can be performed without the use of general anesthesia, only with the use of sedation for chemical containment, plus simple locoregional anesthesia, meeting this procedure’s goals of less invasiveness and lower anesthetic risk [13, 14, 25].

The mean duration obtained for the rumenoscopy-guided rumenostomy procedure was 11.15 ± 0.7 min. Minimally invasive techniques tend to have less surgical time than conventional techniques. In the newly developed video-assisted rumenostomy technique, the time obtained was 13 ± 6.2 min [31], while in conventional techniques, the surgical time varies from 15 to 25 min [33] when performed in a surgical stage. In humans, percutaneous endoscopic gastrostomy was performed in 8 ± 3 min, while the open surgical gastrostomy technique had a mean duration of 35 ± 1.3 min [11].

It is important to highlight that the time in endosurgical techniques depends on the training and experience of the surgeon and the surgical team, as well as on the proper use of the devices to avoid complications and to ensure a good execution of the procedures [9, 21].

The decubitus used in this rumenoscopy technique was efficient, as well as that used in the laparoscopy rumenostomy procedure. In this same procedure, the 36-h fast, combined with the decubitus, allowed a good visualization and manipulation of the rumen, in addition to avoiding regurgitation and inhalation of rumen contents. However, although the percutaneous endoscopic rumenostomy has not yet been tested in live animals, it is believed that the rumenoscopy technique can be performed in standing animals [13, 14, 31].

The visualization of the structures and the possibility of performing the technique in neonate animals using the present study’s model have been confirmed. Further studies are also necessary to verify this technique’s feasibility in adult and larger animals, in which the presence of rumen content, ruminal motility, and larger organ size and length can be complicating factors.

The proposed model has some important points and limitations that require attention when it is eventually tested in living animals. The age of the corpses used, in which the rumen is not yet the main gastric compartment, is one of these factors. The size of the piece and the fact that it is a corpse imply that performing this technique and visualizing the ruminal structures are easier, despite the absence of the peristaltic movements, rumen content, and resistance of the animal to the endoscope.

Franz and Baumgartner, in 2002, used a 100-cm long endoscope for calves up to 7 months of age, and a 150-cm endoscope for animals between 7 months and 6 years of age. The endoscope used in the present study was 100 cm long, which can be a limiting factor for performing the technique in older and larger animals, as it may make it impossible to reach the desired site for cannulation.

Another factor that will need to be evaluated when this technique is tested in live animals is the need for rumenopexy, especially in older and larger animals, due to the weight of the rumen. In 2020, Griffin et al. described a new gastrostomy technique in canine models and highlighted the importance of gastropexy to help prevent complications such as leakage of the stomach contents, especially in dogs weighing more than 25 kg.

As already mentioned earlier, studies involving the cannulation of gastric chambers in ruminants are extremely necessary for animal experimentation, though the clinical need is also a reality [30, 38]. Some specific cases, such as studies on the mitigation of methane produced by ruminants, are extremely important scientific issues today [8, 23].

Thus, the proposed models are the most compatible, because conventional cannulas would interfere with the gas exchange of the rumen with the ambient air, due to cannula displacement, a relatively frequent event [35], as well as at the moment of opening the cannula. These problems were the factors that encouraged our team to develop an in vivo model of minimally invasive rumenostomy in sheep [31], since rumen puncture for many days would bring injury, cannula opening, and ambient air intake, and an oral probe would contaminate the sample with saliva. However, the model can also be executed by new proposed techniques, in simpler and more practical ways.

## Conclusion

The percutaneous endoscopic rumenostomy technique was found to be feasible and efficient when performed in experimental models.

## Methods

As the study corresponds to a new experimental technique, all procedures were performed on cadavers from a locals laughterhouse in accordance with inspection requirements. Tus, the procedures did not cause pain or sufering in animals, as they were performed on bovine fetuses from the slaughtered of pregnant cows. Therefore, is in accordance with Law 11.794 of October 8, 2008, Decree 6899 of July 15, 2009, as well as with the rules issued by the CONCEA, and was approved by the CEUA/UFPA in the meeting of 04/30/2020.

Five anatomical pieces (*n* = 5) were used, corpses of bovine fetuses that were estimated to be between 7 and 9 months of fetal age, resulting from the disposal of a local meat processing company, in which the technique of percutaneous rumenostomy performed by ororuminal endoscopy was used.

The procedure can be better visualized if divided into two stages. The first stage explains the rumenoscopy procedure and the second explains the rumenostomy.

### Rumenoscopy

The corpses were positioned in right lateral decubitus. For the procedure, a flexible endoscope 8.9 mm in diameter and 1100 mm in length was used (Endovision T 190 k, GDI do Brasil, SP, Brazil). The endoscope was introduced through the oral cavity of the fetus, entering the esophagus, where it was necessary to inflate the organ for visualization, using the endoscope’s air pump. The endoscope was introduced until the moment when it was possible to visualize the rumen, which also required being inflated to visualize the ruminal structures and perform the rumenostomy technique. Transabdominal illumination was performed to locate the endoscope end and, by palpation of the flank, establish the exact to insert a 18G catheter mandrel (Fig. 1).

### Rumenostomy

**Fig. 2:**
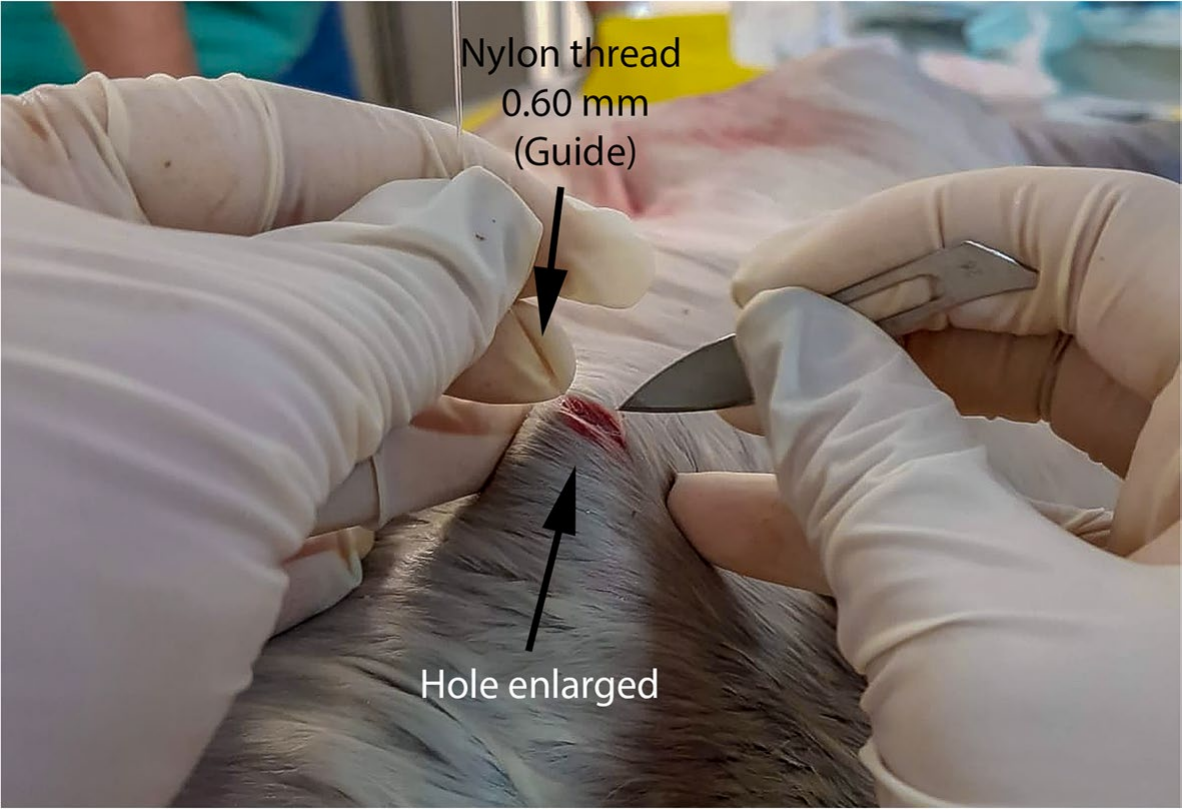
Puncture-incision made to allow the tube exit. Tube’s guidewire

**Fig. 3:**
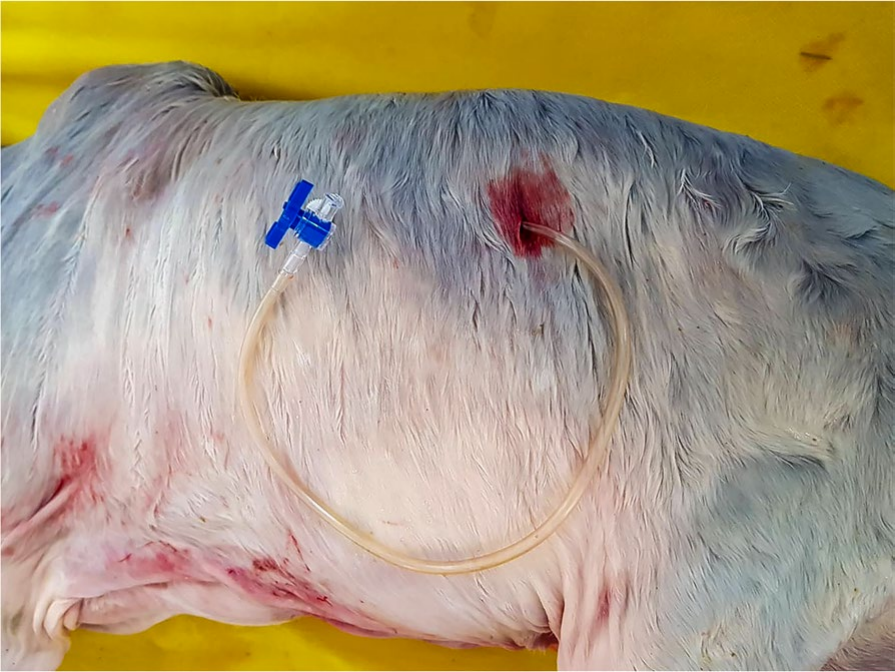
External view of the final positioning of the probe with the three-way stop-cock coupling. Tube used in this tecnique description

**Fig. 4:**
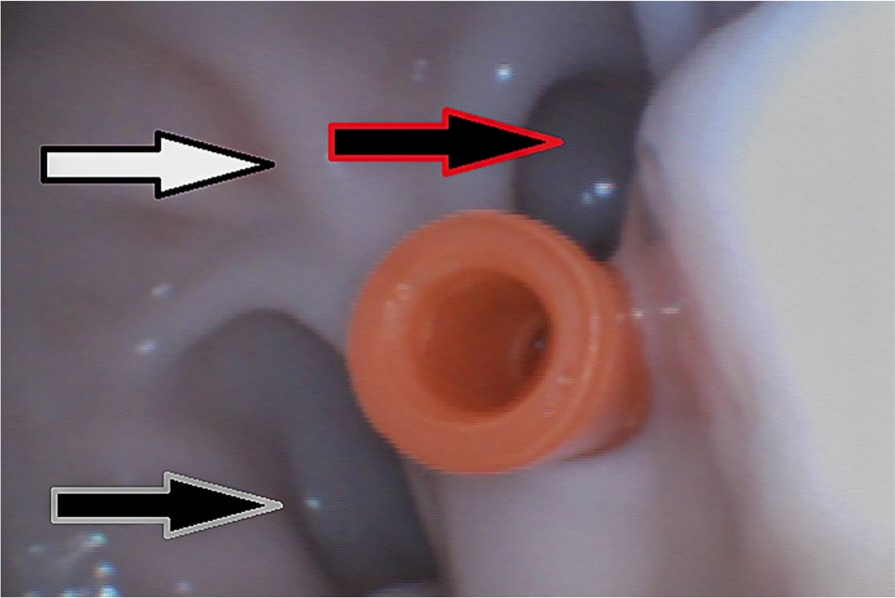
Internal view of the tube at the end of the procedure, where on the white arrow it is possible to observe the caudal dorsal blind sac, signaled by the yellow arrow, the caudal pillar can be seen, and pointed by the black arrow the caudal ventral blind sac. Three-way stop-cock coupling

Once the site of insertion of the mandrel was established, the catheter mandrel was inserted and the guide probe was passed through it, using 0.60-mm nylon thread, with the length varying according to the size of the fetus (Fig. 1A). The guide was seized by the endoscopic grasping forceps alligator jaws, which was passed through the endoscope’s working channel. Then the guide was taken to the mouth of the experimental piece and the outer tip of a size 14 gastric probe was fixed to the guide. The guide was then moved to the inside of the rumen and then to the point of insertion of the guide on the flank. By tractioning the guide, the entrance hole was enlarged with scalpel just enough for the probe to exit (Fig. 2). After that, the probe was sutured using the Chinese knot stitch technique and coupled to a three-way valve to manage content collection and air intake in the rumen (Figs. 3 and 4).

The entire procedure was timed and thedata on the visualization of ruminal structures, decubitus, inflation, illumination, and probe positioning were described, and the average procedure time was obtained.

## Supplementary Information

**Additional file 1.**

## Data Availability

All data generated or analysed during this study are included in this published article [and its supplementary information files].
